# Cyber War Game in Temporal Networks

**DOI:** 10.1371/journal.pone.0148674

**Published:** 2016-02-09

**Authors:** Jin-Hee Cho, Jianxi Gao

**Affiliations:** 1 Computational and Information Sciences Directorate, U.S. Army Research Laboratory, Adelphi, MD 20783, United States of America; 2 Center for Complex Network Research and Department of Physics, Northeastern University, Boston, Massachusetts 02115, United States of America; Beihang University, CHINA

## Abstract

In a cyber war game where a network is fully distributed and characterized by resource constraints and high dynamics, attackers or defenders often face a situation that may require optimal strategies to win the game with minimum effort. Given the system goal states of attackers and defenders, we study what strategies attackers or defenders can take to reach their respective system goal state (i.e., winning system state) with minimum resource consumption. However, due to the dynamics of a network caused by a node’s mobility, failure or its resource depletion over time or action(s), this optimization problem becomes NP-complete. We propose two heuristic strategies in a greedy manner based on a node’s two characteristics: resource level and influence based on *k*-hop reachability. We analyze complexity and optimality of each algorithm compared to optimal solutions for a small-scale static network. Further, we conduct a comprehensive experimental study for a large-scale temporal network to investigate best strategies, given a different environmental setting of network temporality and density. We demonstrate the performance of each strategy under various scenarios of attacker/defender strategies in terms of win probability, resource consumption, and system vulnerability.

## Introduction

Many natural and man-made systems can be modeled as complex networks consisting of nodes and links representing the interactions between nodes [1, 2]. One of the most important property of a network is robustness against random failures and target attacks [3–7], measured by the giant connected component size after perturbations. The percolation threshold is the fraction of non-removed nodes (or links) leading to the collapse of the network [1, 4], which is often predicted by using percolation theory, a method from statistical physics [1, 8]. Increasing evidence shows that networks interact to each other, resulting in a new research area on interdependent networks [9, 10], interconnected networks [11], multiplex [12], multilayer networks [13], and a network of networks [14, 15]. Indeed, these systems can not only model interactions between different networks, but also consider a temporal network [16] in which a network topology changes over time. Understanding vulnerability of these systems helps design interdependent robust infrastructures.

Unlike engineering systems, vulnerability of temporal, mobile networks can be modeled as the cyber games where the attackers intend to compromise users and and the defenders will recover the compromised users to healthy state under the nodes’ resource restriction such as battery life, computational power, and/or the network’s limited bandwidth [17]. Furthermore, an entity often requires decision making based on local information in a fully distributed way and aims to take optimal strategies to maximize resource efficiency (e.g., complete a task with minimum effort) when achieving respective goals. For instance, an attacker compromises more healthy nodes to disrupt a system while a defender recovers compromised nodes to secure the system. Although many existing approaches consider cyber war games by proposing optimal strategies of attackers and defenders [17–20], they do not consider optimal strategies with minimum resource consumption in temporal networks.

An attacker-defender cyber game has been explored with various approaches such as game theory [18] or cognitive theory [20]. Zhu and Martinez [18] model a cyber game using a two-level Stackelberg game (leader-follower) to consider a node’s inherent resource constraints in discrete-time, linear time-invariant networks. Recently, Ben-Asher and Gonzalez [20] propose a decision making framework using an instance-based learning technique considering dynamics of a cyber war where multiple attackers and defenders play to maximize their utility. In distributed cyberspaces, however, a network suffers from resource constraints and faces high dynamics under varying network temporality and density. In this work, we question a fundamental problem: how does the network temporality affect the performance of attackers or defenders in a cyber war game under resource-constrained, distributed network environments?

To answer this question, we aim to identify optimal strategies of attackers or defenders that allow a winning in a cyber game with minimum resource consumption in a time-varying, distributed network. In this environment, each node has a limited resource and its resource level is updated over time or upon taking actions. Due to the distributed nature of a network, a node may use local information to make decisions and often can take actions towards its adjacent nodes. That is, attackers or defenders may select a node to compromise or recover among their adjacent nodes, respectively. Considering these challenges derived from the unique characteristics of a given network environment, this optimization problem is not solvable in a polynomial time and known as a NP-complete problem. This work has the following unique contributions:

1. We consider resource efficiency of cyber strategies taken by attackers or defenders in a resource-constrained, distributed network environment where each attacker or defender can make a decision based on local information without the knowledge of global network (e.g., network topology) and node information (e.g., remaining energy).
2. We consider a time-varying network such as structural and state dynamics and study how they affect the optimal strategies. Structural dynamic refers to network topology changes that may be caused by node mobility or failure or terrains while state dynamic means resource depletion over time or upon action(s). To consider structural dynamics, we introduce a new influence metric called *k*-hop influence based on the concept of *k*-hop reachability. For state dynamics, we consider dynamic adjustment of each node’s status. Both dynamics affect decisions by attackers or defenders to reach their respective system goal state.
3. We conduct comprehensive performance analysis of the proposed strategies by attackers or defenders which studies the impact of network temporality and density on our performance metrics such as a win probability, minimum resource consumption, and system vulnerability.

## System Model

In this section, we explain our network model, node model, and system failure condition considered in this work.

### Network Model

We consider a temporal network whose topology changes over time. In our model, at an initial time we generate a random arbitrary network using a given degree distribution. Every time step we randomly select *p* fraction of link and rewire the nodes between these links randomly. When *p* = 0, the network is static; when *p* = 1, we generate a new random network independent from the previous step. In addition, nodes’ resource level depletes with more actions and over time. Given a network with a directed graph G(t)=(V,ℰ(t):W(t)) at time step *t*, V(t) is a set of vertices, representing nodes (or entities) and ℰ(t) is a set of edges, representing connectivity between two vertices. Depending on the existence of an edge between two nodes, *i* and *j*, the weight *w*_*ij*_(*t*) can be in W(t), i.e., $w_{ij}(t)\in W(t)$.

A given network environment is characterized by: (1) it is highly distributed where each node can communicate with or take an action towards its adjacent node(s); (2) it is severely resource-constrained where a node may drain its resource (i.e., battery life or reliability) over time to maintain normal operations even without interacting with other nodes or may consume resource when it takes an action towards any adjacent node; and (3) it is time-varying, dynamic in terms of network temporality (i.e., changing network topology) and remaining resource level of nodes.

**Fig 1:**
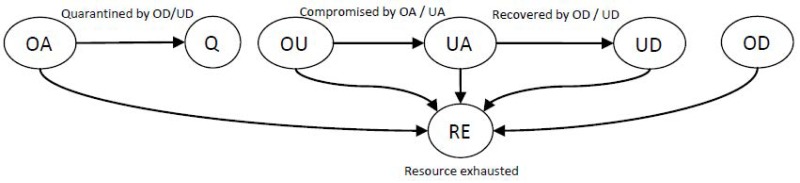
Composition of nodes and their dynamic status. OA for original attackers, Q for quarantined original attackers, OU for original users which have never compromised or recovered before, UA for compromised users becoming attackers, UD for recovered users becoming defenders, OD for original defenders, and RE for resource exhausted. All nodes except the quarantined attackers are regarded as legitimate member nodes and can become resource-exhausted, resulting in non-legitimate members. Where N=|C(t)|+|D(t)|+|A(t)|+|ℐA(t)|, we can derive D(t)=OD(t)∪UD(t), C(t)=OA(t)∪UA(t), A(t)=U(t), and ℐA(t)=Q(t)∪RE(t) at time *t* > 0.

We use epidemic spreading based on susceptible-infected-removed (SIR) model [6, 21] to describe attackers’ compromising behaviors and defenders’ recovering behaviors. If a node is recovered from being compromised, it is immune to the attack. Thus, in terms of a node’s lifetime except original attackers or defenders seeded in the network deployment, a user node only experiences one time to be compromised by attackers or recovered by defenders. A network is initialized with three types of players including attackers, defenders, and users based on their state (see Fig 1). We assume that a node is equipped with a host-based intrusion detection mechanism [22], characterized by probabilities of false positives and false negatives, denoted as *P*_*fp*_ and *P*_*fn*_, respectively. Each node *i* is capable of extrapolating neighboring nodes *j*’s resource level based on their activities and signal strength. For each node to be aware of partial or complete network topology, a node broadcasts its neighbor information to the network. Obtaining a global network topology requires all nodes’ neighboring information which is heavily expensive in resource-constrained environments (e.g., wireless mobile networks). To mitigate this overhead, we introduce the concept of *k*-hop reachability, by limiting the geographic area of disseminating information of adjacent nodes.

We consider two types of dynamics associated with a node’s characteristics in terms of structural dynamic and state dynamic as follows:

1. Structural dynamic is reflected based on a node’s temporal location in a network, related to the work on network of networks [23–25]. We investigate a node’s influence based on the concept of *k*-hop reachability [26] in a given time-varying network and employ it as criteria for an attacker’s or defender’s decision to select a node to take an action (i.e., compromising or recovering a node). We explain the computation of a node’s influence in Eq (3) below.
2. State dynamic is considered in terms of a node’s resource level representing battery life and/or reliability. Structural and state dynamics may evolve at the same time [27–29]. The structural and state dynamics are interwoven and affect to each other, the so called dynamics of mutualistic interactions [27]. For example, species abundance affects network rewiring while network structure determines the species abundance. In addition, the coevolution of state and structural dynamics leads to the nestedness of real mutualistic networks [28]. Other examples can be observed in a collective motion of self-propelled particle systems [28] or a network of self-propelled agent systems [29]. In our work, a network topology partially relies on state dynamic while the state dynamic fully depends on structural dynamic.

### Node Model

Recall that a given cyber war game is played by attackers, defenders, and users. When an attacker compromises a user, the user is compromised and becomes an attacker who is capable of attacking another healthy user (i.e., a node which has not compromised in the past). Unless the compromised user is recovered by a defender, it remains as compromised. A defender is a node with the capability to recover a compromised user or to quarantine an original attacker. If a compromised user is recovered by the defender, it is immune to the attack such as SIR [6, 30–32]. If the recovered node is an original attacker, it is quarantined and cannot perform any attack while it does not have a capability to recover another node. The defender is robust against attacks and will never be compromised by an attacker in which the defenders are trusted entities. Winning a given cyber war depends on whether or not attackers or defenders reach their system goal state, respectively.

Given an initial number of nodes N in a network, the network has the following four types of nodes at time *t* based on their state:

1. Compromised nodes (C(t)) include original attackers or compromised users;
2. Healthy active user nodes (A(t)) are users who have never compromised in the past;
3. Defenders (D(t)) indicate original defenders or recovered users; and
4. Inactive nodes (ℐA(t)) are dead nodes due to lack of resources or original attackers being quarantined.

The total number of nodes initially given N can be derived as N=|C(t)|+|D(t)|+|A(t)|+|ℐA(t)|. We summarize the network node composition and dynamic status of nodes in Fig 1.

In a given network, a certain fraction of nodes are compromised by outside attacker(s). We study how quickly attackers or defenders reach their respective goal state from the initial state as shown in Fig 1. As time elapses, the compromised nodes start compromising other legitimate member nodes based on their attack strategy to reach the system failure state based on System Failure Condition (SFC) (see a next subsection, System Failure Condition). We discuss attacker and defender strategies in Section Attacker and Defender Strategies later. As some nodes become compromised, defenders detect them and start performing the recovery process of compromised nodes to prevent or mitigate system failure by eliminating all compromised nodes from the system.

Next we represent the characteristics of a node as a vector by:

$$v_{i}\left(t\right)={\left[r_{i}\left(t\right),d_{i,in}^{\left(k\right)}\left(t\right),d_{i,out}^{\left(k\right)}\left(t\right)\right]}^{T}$$ (1)

where *r*_*i*_(*t*) is node *i*’s resource level at time *t* and $d_{i,in}^{\left(k\right)}(t)$ and $d_{i,out}^{\left(k\right)}(t)$ indicate the in-degree and out-degree of node *i* within *k*-hop distance from itself at time *t*, respectively. The out-degree of node *i* with a given *k* indicates the concept of reachability, i.e., how many nodes are reachable from node *i* in a network. The in-degree of node *i* with *k*-hop distance means how many nodes can reach node *i* within *k*-hop distance. We use *k*-hop reachability [26] to mitigate the computation or communication overhead to exchange neighbors information. We use the *k*-hop distance in-degree and out-degree of node *i* to calculate its influence in the network.

Each node disseminates adjacent nodes information in order to provide a global view of the network. To mitigate high communication overhead, it disseminates neighbors information within a *k*-hop distance. Given an adjacency matrix, W(t), for a directed graph G(t)=(V(t),ℰ(t)), matrix *L*^(*k*)^(*t*) consists of elements $l_{ij}^{k}(t)$ with a binary value 0 or 1 representing that node *j* is reachable from node *i* within *k*-hop distance. *L*^(*k*)^(*t*) is computed based on the shortest path algorithm for a directed graph considered [33].

Based on *L*^(*k*)^(*t*) calculated above, let *D*^(*k*)^(*t*) be a 2 × *n* matrix for the in-degree and out-degree of *n* number of nodes based on *k*-hop distance. *D*^(*k*)^(*t*) is denoted as each element with $d_{i,in}^{\left(k\right)}(t)$ and $d_{i,out}^{\left(k\right)}(t)$, which are calculated based on *L*^(*k*)^(*t*) with elements $l_{ij}^{k}(t)$ for all *i* and *j* by:

$$d_{i,in}^{\left(k\right)}\left(t\right)=\sum_{j=1,l_{ij}^{k}\neq \infty }^{n}l_{ij}^{k}\left(t\right),\;d_{i,out}^{\left(k\right)}\left(t\right)=\sum_{j=1,l_{ji}^{k}\neq \infty }^{n}l_{ji}^{k}\left(t\right)$$ (2)

The degree of a node’s influence is used as one of criteria attackers or defenders take actions to minimize the accumulated resource consumption until they reach the respective system goal state. A node’s influence is calculated by:

$$I_{i}^{k}\left(t\right)=\frac{d_{i,out}^{\left(k\right)}\left(t\right)}{d_{i,in}^{\left(1\right)}\left(t\right)}$$ (3)

$I_{i}^{k}(t)$ implies a node’s influence over other nodes in a network with a given *k*-hop distance compared to other nodes’ influence over the node itself. A node with high influence, $I_{i}^{k}(t)$, means that the node has high influence over others while it is not much influenced by other nodes. For simplicity, we did not include time unit *t* in the equations above but the influence may be affected by the dynamics of a network topology which was examined in the simulation experiments by varying network temporality.

We consider the state *r*_*i*_(*t*) of a node *i* changes over time according to its incoming neighboring nodes, strategy chosen, and whether an action is taken or not. *r*_*i*_(*t*) is *i*’s remaining resource level at time *t*. Based on *e*_*i*_(*t*) above, node *i*’s remaining resource level, *r*_*i*_(*t*), is updated as:

$$r_{i}\left(t\right)=\left\{\begin{array}{c} r_{i}\left(t-1\right)-\sigma -e_{i}\left(t\right)\;\;\text{if}\;i\;\text{takes}\;\text{an}\;\text{action;}\\ r_{i}\left(t-1\right)-\sigma \;\;\;\;\;\;\;\;\;\;\;\;\;\;\text{otherwise} \end{array}\right.$$ (4)

where *r*_*i*_(*t* = 0) for all *i*’s is randomly selected as a real number ranged in [0.5, 1] based on uniform distribution and its resource level, *r*_*i*_(*t*), decreases over time. *σ* denotes a decay of resource over time to maintain its normal operation, ranged in [0, 1] as a real number. *e*_*i*_(*t*) is defined as:

$$e_{i}\left(t\right)=\left\{\begin{array}{c} \frac{\lambda r_{j}\left(t\right)}{r_{i}\left(t\right)}\;\;\text{if}\;i\;\text{selects}\;j\;\text{to}\;\text{take}\;\text{an}\;\text{action;}\\ 0\;\;\;\;\;\;\;\;\;\text{otherwise} \end{array}\right.$$ (5)

*e*_*i*_(*t*) counts the cost only when node *i* chooses node *j* to take an action; 0 otherwise. The above implies that when node *j* has a high resource level, an attacker or a defender needs to consume more resource to take an action towards node *j*. *λ* is a constant parameter to adjust the speed of the resource consumption per action. *e*_*i*_(*t*) implies node *i* consumes more resource to take an action towards node *j* with higher resource level. Note that node *i* takes an action only when *r*_*i*_(*t* − 1) − *σ* − *e*_*i*_(*t*)>0.

When node *i* selects node *j* to take an action, node *i*’s action is effective towards node *j* with a probability by:

$$s_{ij}\left(t\right)=\text{min}\left[\frac{r_{j}\left(t\right)}{r_{i}\left(t\right)},1\right]$$ (6)

*s*_*ij*_(*t*) implies that when node *j* has high resource, node *i*’s action is less likely to be effective, vice-versa.

When *r*_*i*_(*t*) = 0, it means node *i* dies due to the lack of resource. This node is not part of legitimate members in the network, and accordingly the total number of active nodes at time *t*, N(t), decreases. ℐA(t) increments as more inactive nodes exist in the network. In this work, each attacker or defender can compromise or recover one adjacent node at a time, not allowing actions towards multiple nodes simultaneously.

### System Failure Condition

The goal of attackers is to reach the system state to failure. To model the attackers’ target state based on the system failure state, we define the system failure condition (SFC) in terms of the loss of two system security goals: (1) loss of integrity based on the concept of Byzantine Failure [34] where the system fails with too many compromised entities (e.g., the system with more than one-third of participating entities being compromised), leading to increased attack severity due to collusive attack; and (2) loss of availability based on the fact that the system does not have a sufficient number of healthy, active nodes for mission execution. Some nodes may die due to lack of resources while other nodes may be compromised due to node capture attack by attackers. Therefore, the SFC is defined by:

$$SFC=\left\{\begin{array}{c} 1\;\text{if}\;\frac{\left|C(t)\right|}{N(t)}\geq \rho_{1}⋁\frac{\left|C(t)|+|ℐA(t)\right|}{N}\geq \rho_{2}\\ 0\;\text{otherwise;}\; \end{array}\right.$$ (7)

where |C(t)| is the total number of compromised nodes at time *t*, N(t) refers to the number of active nodes at time *t* regardless of their status, either compromised or healthy. |C(t)|+|ℐA(t)| indicates the total number of inactive nodes including original attackers quarantined plus dead user nodes due to lack of resource. Where N is the total number of nodes that are initially given, *ρ*_1_ bounds the maximum number of compromised nodes that can exist without failure while *ρ*_2_ is the fraction of the maximum number of inactive nodes that can exist without failure in the network.

## Cyber War Game

This section discusses how the cyber war game is formulated as an optimization problem. In addition, we describe attacker and defender strategies proposed in this work and analyze their solution complexity.

### Problem Formulation

We formalize this problem as an optimization problem that minimizes accumulated resource consumption J until the system goal state reaches by solving the following objective function as:

$$\begin{array}{l} \text{Minimize}\;J=\overset{T}{\underset{t=0}{\int }}\underset{i\in ℳ\left(t\right)}{\sum }e_{i}\left(t\right)dt\\ \text{Subject}\;\text{to}\;w_{ij}\left(t\right)>0,r_{i}\left(t\right)-e_{i}\left(t\right)>0 \end{array}$$ (8)

Here ℳ(t) is a set of nodes belonging to a party (i.e., either attackers or defenders) where ℳ(t) includes a set of nodes taking actions to reach a respective system target state. ℳ(t) is same as C(t) for attackers while it is D(t) for defenders in Fig 1.

For attackers, **e**(*t*) is a vector of the resource consumed by attackers successfully where **e**(**t**) = [*e*_1_(*t*), …, *e*_*i*_(*t*), …, *e*_*m*_(*t*)]^*T*^ and m=|C(t)| and *e*_*i*_(*t*) represents resource consumed by node *i* to compromise another node in U(t) at time *t* (See Eq (5) for *e*_*i*_(*t*)). Similarly, for defenders, **e**(**t**) is a vector of the resource consumed by the defenders to successfully recover compromised nodes in C(t), where **e**(**t**) = [*e*_1_(*t*), …, *e*_*i*_(*t*), …, *e*_*m*_(*t*)]^*T*^ and m=|D(t)| and *e*_*i*_(*t*) indicates node *i*’s resource consumption to recover a compromised node at time *t*. This problem is to identify a set of nodes by which attackers or defenders take actions to reach their respective goal state while minimizing resource consumption. Recall that attackers or defenders can only take actions towards their adjacent neighbors (i.e., 1-hop neighbor).

In Eq (8) above, a small amount of resource decay over time (i.e., *σ*) without any additional activity (e.g., compromising or recovering actions) is omitted. The imposed constraints are: (1) node *i* can take an action towards node *j* only when *w*_*ij*_(*t*)>0 which means there is a directed edge from node *i* to node *j*; and (2) node *i* should have sufficient resource to take an action towards node *j* (i.e., *r*_*i*_(*t*) − *e*_*i*_(*t*)>0).

### Attacker and Defender Strategies

In this section, we discuss what strategies attackers or defenders can take to win a cyber war game with minimum resource consumption, respectively.

Node *i*, either attacker or defender, selects an adjacent node *j* (it should be originally a user) with minimum resource consumption to compromise or recover node *j* while reaching the target state as quickly as possible. We propose two heuristic strategies based on a node’s two characteristics as follows: (1) a node’s resource level; and (2) a node’s influence based on *k*-hop reachability as shown in Eq (3), called *k*-hop influence in this work. Therefore, each node *i* can have two strategies to select adjacent node *j* to take an action as follows:

1. *Resource-First* (RF): node *i* selects node *j* with the minimum resource among all adjacent nodes.
2. *Influence-First* (IF): node *i* selects node *j* with the maximum influence among all adjacent nodes.

Both strategies above have the goal to win a game with the minimum resource consumption by either minimizing resource consumption in each step or maximizing a chance to reach the goal state with minimum time where both strategies aim to minimize the accumulated resource consumption until the end state. We denote attackers’ two strategies as Resource-First-Attack (RF-A) and Influence-First-Attack (IF-A). Similarly, defenders’ strategies are notated as Resource-First-Defense (RF-D) and Influence-First-Defense (IF-D). In all cases, if node *i*’s expected resource consumption by taking an action towards node *j* exceeds its current remaining resource in Eq (4), node *i* does not take any action towards node *j* to save its resource for its own survival.

### Solution Complexity Analysis

In this section, we analyze the solution complexity of three algorithms: optimal solution using brute-force algorithm (BFA) based on depth-first-search, resource-first (RF) and influence-first (IF). In particular, we analyze the strategies taken by attackers and how their strategies affect the resource consumption. In order to find feasible solution space, we relax some conditions defined in this work. The time-varying network condition is relaxed by using a static network to identify optimal solution using BFA. We assume that a static network has nodes connected to other nodes with a weight *e*_*ij*_ where *e*_*ij*_ is computed based on *e*_*i*_ in Eq (5) where an edge exists between nodes *i* and *j*. Note that *e*_*ij*_ is updated whenever node *i* takes an action with any adjacent node. For example, *i*’s resource will be updated as it takes actions towards adjacent nodes *j*’s. Accordingly, *e*_*ij*_ for all adjacent nodes *j*’s is affected by node *i*’s resource adjustment. In order to minimize the effect of randomness, we consider *s*_*ij*_ = 1. Note that we remove all the relaxed conditions used above and show simulation results for a large time-varying, dynamic network later in a next section.

In this section, we analyze the complexity of solution search in three algorithms including BFA, RF and IF. We analyze the complexity in terms of attackers’ perspective where the attackers require compromising more nodes to reach their goal state as defenders recover the compromised nodes over time. We approximate the complexity of solution search algorithms where a graph has *n* vertices and each vertex has an average of *m* out-degrees.

1. Brute Force Attack (BFA): we simply calculate the combination of choosing *c* out of *n* where *n* is the total number of nodes and *c* is the number of nodes required to be compromised to meet SFC. Since the loss of integrity failure is a more tight condition than the loss of availability failure unless many nodes quickly drain their resources, we treat *c* as the minimum number of compromised nodes to make the system failure in this case. This is computed by selecting *c* number of compromised nodes out of *n* which is the initial number of nodes given, denoted as *C*(*n*, *c*) = *O*(*n*2^*n*^) where *c* ≤ *n*/2 (i.e., *ρ*_1_ ≤ 1/2) and *n*! = *o*(*n*^*n*^). Since each node computes this brute-force solution, resulting in *O*(*n*2^*n*^) and there is the overhead to obtain global network topology, *O*(*n*^3^), the complexity of brute-force optimal solution is *O*(*n*2^*n*^+*n*^3^), leading to *O*(*n*2^*n*^).
2. Resource-First-Attack (RF-A): it is linear proportional to *n* as each attacker chooses one among multiple adjacent nodes based on the minimum resource. Given an initial number of attackers *c*_0_, and the average out-degrees *m*, the maximum round of compromising actions by all attackers, *h*, each attacker compromises another healthy node. It is estimated by:
  $$\begin{array}{c} 2^{0}mc_{0}+2^{1}mc_{0}+2^{2}mc_{0}+...+2^{h}mc_{0}=\sum_{i=0}^{h}2^{i}mc_{0}<h2^{h}mc_{0}\\ \text{where}\;2^{h}mc_{0}<n<hn=O\left(n\right)\;\text{for}\;h<<n \end{array}$$
  Therefore, RF-A has a complexity of *O*(*n*^2^) where each node runs *O*(*n*).
3. *Influence-First-Attack (IF-A):* it is similar to RF-A for the compromising process, *O*(*n*^2^), but it has the overhead to compute *k*-influence, leading to *O*(*n*^3^).

## Experiments and Analysis

We show the results and analyze their trends under two network environments: (1) a static network to identify optimal solutions; and (2) a temporal network by varying network temporality *p* and nodes’ average degree *d*.

### Metrics

The following performance metrics are used:

1. Win probability ($P_{w}$) refers to attackers’ average win probability. Attackers win when SFC is met; defenders win when there exist no compromised nodes in a network.
2. Resource Consumption(J) is the average accumulated resource consumed by either attackers or defenders until time *T* which is the time that they attain their respective goal based on Eq (8).
3. System Vulnerability (V(t)) refers to the degree of the system vulnerability in terms of the number of compromised nodes and the number of inactive nodes, as addressed in SFC. When V(t)≥1, this indicates the system failure state. This is estimated by: n
  $$V\left(t\right)=\text{max}\left[\frac{\left|C(t)\right|}{\rho_{1}N\left(t\right)},\frac{\left|C(t)|+|ℐA(t)\right|}{\rho_{2}N}\right]$$ (9)
  where $\rho_{1}N(t)$ is the maximum number of compromised nodes allowed in the system without failure at time *t* and |C(t)| is the number of compromised nodes in the system at time *t*. $\rho_{2}N$ indicates the maximum number of members that are not committing for mission execution and is the maximum bound tolerated by the system. |C(t)|+|IA(t)| is the number of inactive or compromised members currently in the network.

### Result Analysis under a Small-Scale Static Network

#### Experimental Setting

Since it is not feasible to obtain optimal solution(s) of a given problem under a time-varying network consisting of a large number of nodes (i.e., NP-Complete), we first validate the optimality of the given problem by comparing the three algorithms in a static network consisting of 20 nodes. In the next section below, we will discuss results under temporal networks. We use an environment setting with *λ* = 0.05, *σ* = 0.001 (i.e., in resource calculation in Eq (4)), and *k* = 2 (i.e., in *k*-hop influence in Eq (3)). Resource levels of nodes are assigned as a real number ranged in [0.5, 1] based on the uniform distribution in order to consider vastly different resource levels of nodes in a network. We set up the network based on Erdös-Rényi (ER) model with a different probability, *q*, given for two nodes to be randomly connected in the network deployment. To make the given network have vastly different degrees in a directed network, we randomly select a pair of nodes to remove an edge (i.e., when edges exist between nodes *i* and *j* in both directions such as *i* to *j* or vice-versa, we keep one edge while removing the other edge).

#### Result

**Fig 2:**
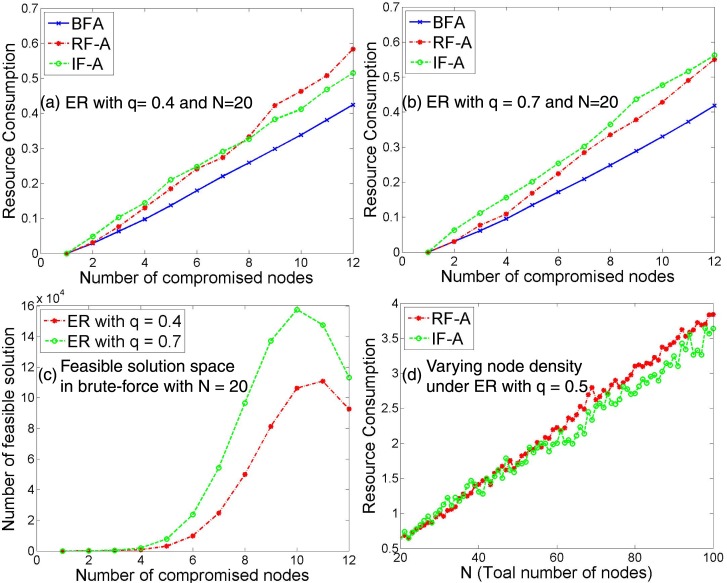
Optimality Analysis of Attack Strategies in a Static Network. (a)-(b) For ER networks composed of *N* = 20 nodes with (a) *q* = 0.4, and (b) *q* = 0.7, we plot the resource consumption as a function of the number of compromised nodes for three different strategies. (c) Number of feasible solution as a function of the number of compromised nodes for two different ER networks. (d) Resource consumption as a function of the number of nodes for two different strategies.

In Fig 2, we show the minimum resource consumed by three algorithms including two strategies and one optimal solution based on BFA. In order to demonstrate the optimality of the given problem, we use a simplified cyber war scenario. The scenario is that an attacker compromises user nodes while a defender may recover compromised user nodes. Thus, in Fig 2, we show the number of compromised nodes as x-axis while plotting the resource consumption in y-axis. Each strategy shown here is a strategy taken by an attacker, such as brute-force-attack (BFA), resource-first-attack (RF-A), or influence-first-attack (IF-A).

As shown in Fig 2(a) and 2(b), although BFA performs the best consuming the minimum resource among three in both network conditions (i.e., sparse with *q* = 0.4 and dense with *q* = 0.7), it occurs prohibitively high overhead for solution search as shown in Fig 2(c). For the other two strategies, RF-A or IF-A, we notice IF-A becomes outperforming RF-A as attackers compromise more nodes particularly under a sparse network. Lastly we experiment the impact of varying the total number of nodes, N, in a network. In Fig 2(d), we investigate the impact of N on resource consumption of the two strategies. We set the number of compromised nodes to N×2/3. In this case, IF-A outperforms RF-A as a network size becomes larger. In a network with higher node density but less network density (i.e., sparse with *q* = 0.5 in this case), attackers prefer IF-A over RF-A in compromising a large number of nodes in the network.

### Result Analysis under a Large-Scale Temporal Network

#### Experimental Setting

For a large-scale temporal network, we use a random network based on ER network model where network temporality *p* is used as a rewiring probability that two nodes *i* and *j* are connected at time *t*. We consider the total number of nodes, N=1000 where the nodes consists of the initial number of attacker, |*OA*| = 1, the initial number of defenders, |*OD*| = 50, and the initial number of users, |*OU*| = 949. We set *λ* = 0.05, *σ* = 0.001, and *k* = 6. For network environmental conditions, network temporality *p* and nodes’ average degree *d* (i.e., higher *d* indicates higher network density) are varied to observe their impact on performance. All data points shown in the results are collected based on 100 of realizations. We summarize all key design parameters, their meanings and corresponding default values in Table 1. For dependent variables, we note dependent under ‘Value’ in Table 1.

**Table 1:** Key design parameters, their meanings and default values.

**Param.**	**Meaning**	**Value**
N	Number of nodes deployed in a network	1000
*σ*	Decay of resource over time to maintain its normal operations ranged in [0, 1]	0.001
*λ*	A constant parameter value to adjust the speed of the resource consumption per action	0.05
*P*_*fp*_, *P*_*fn*_	False positives and false negatives probabilities of a host-based IDS preinstalled in each node	0.05
*ρ*_1_	Fraction used to determine the maximum number of compromised nodes allowed in the system without failure	1/3
*ρ*_2_	Fraction used to determine the maximum number of members that are not committing for mission execution	2/3
*k*	Number of distance hops to consider *k*-hop reachability	6
|*OA*|	Initial number of attackers	1
|*OD*|	Initial number of defenders	50
*Q*(*t*)	Quarantined original attackers at time *t*	dependent
*UD*(*t*)	A set of recovered users becoming defenders at time *t*	dependent
*RE*(*t*)	A set of nodes with resource exhausted at time *t*	dependent
N(t)	A set of active nodes in a network at time *t*	dependent
C(t)	A set of compromised nodes in a network at time *t*	dependent
D(t)	A set of defender nodes in a network at time *t*	dependent
IA(t)	A set of of inactive nodes in a network at time *t*	dependent
A(t)	A set of healthy active nodes in a network at time *t*	dependent
*v_i_*(*t*)	A vector of a node’s state at time *t* in terms of resource consumption, in-degree and out-degree	dependent
*r*_*i*_(*t*)	Node *i*’s remaining resource at time *t*	dependent
$d_{i,in}^{\left(k\right)}$	Node *i*’s in-degree using *k*-hop reachability	dependent
$d_{i,out}^{\left(k\right)}$	Node *i*’s in-degree using *k*-hop reachability	dependent
**e(t)**	A vector of resource consumed by attackers or defenders taking actions	dependent
*e*_*i*_(*t*)	Resource consumed when node *i* takes an action towards node *j*	dependent
*s*_*ij*_(*t*)	Probability that node *i*’s action is effective against node *j* at time *t*	dependent

#### Result

**Fig 3:**
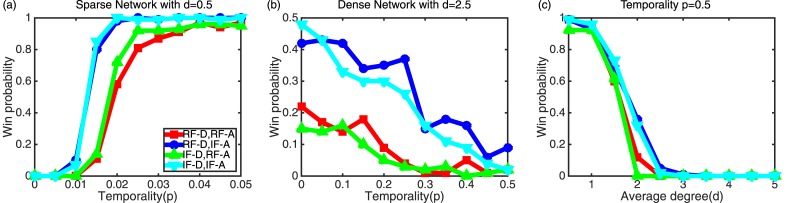
Effect of network temporality (*p*) and density (*d*) on win probability (*P*_*w*_). (a) A win probability as a function of temporality for four pairs of strategies by defenders and attackers under a sparse network with average degree *d* = 0.5. (b) A win probability vs. temporality for four pairs of strategies of defenders and attackers under a dense network with average degree *d* = 2.5. (c) A win probability as a function of an average degree for four pairs of strategies of defenders and attackers with high network temporality *p* = 0.5.

Fig 3 shows how network temporality *p* and network density *d* affect attackers’ win probability. We vary *p* to see its impact on *P*_*w*_ under sparse or dense network with *d* = 0.5 or *d* = 2.5, respectively, as shown in Fig 3(a) and 3(b). In a sparse network of Fig 3(a), regardless of defenders’ strategies, attackers’ IF outperforms among others. In addition, higher *p* in a sparse network helps attackers to win. In a dense network of Fig 3(b), although attackers’ IF outperforms the RF counterpart, when defenders choose RF, attackers have higher chances to win the game. More interestingly, in this dense network, higher network temporality *p* deteriorates attackers’ chances to win. Fig 3(c) shows the effect of varying *d* on attackers’ *P*_*w*_. In all strategy scenarios, higher *d* does not help attackers to win because higher network density will increase a chance for a node to be recovered by defenders.

**Fig 4:**
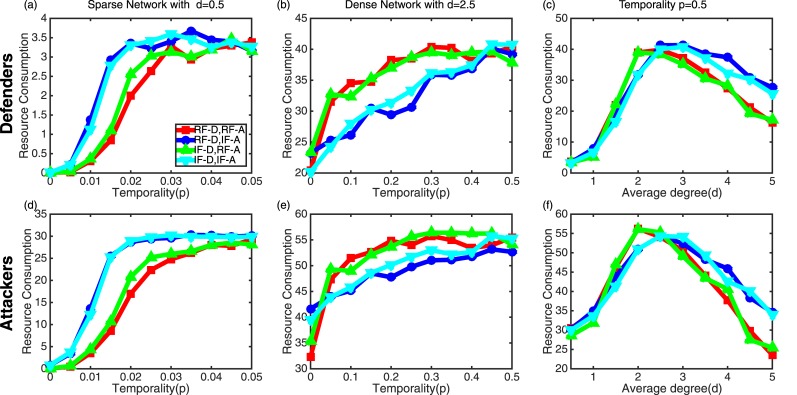
Effect of network temporality (*p*) and density (*d*) on defenders’ and attackers’ resource consumption (J). (a) Resource consumption of defenders vs. temporality under a sparse network with average degree *d* = 0.5. (b) Resource consumption of defenders vs. temporality under a dense network with average degree *d* = 2.5 (b). (c) Resource consumption of defenders as a function of average degree for four pairs of strategies of defenders and attackers with high network temporality *p* = 0.5. (d)-(f) Similar plots for attackers’ resource consumption.

Fig 4 shows how network temporality and density impact attackers’ resource consumption (J). In a sparse network of Fig 4(a), higher J occurs as *p* increases and when attackers take IF strategy. Higher *P*_*w*_ leads to higher J because attackers should take more actions. For a dense network of Fig 4(b), higher *P*_*w*_ does not necessarily lead to higher J. This case is shown when attackers use RF (i.e., red and green curves). This is because choosing a node with low remaining resource does not lead to a higher chance to compromise more nodes in next decision rounds. In Fig 4(c), a critical point of *d* exists in maximizing J. A network with smaller *d* helps attackers to win quickly due to a less chance to be recovered by defenders in a sparse network. On the other hand, a dense network with high *d* allows compromised nodes to be easily recovered. Thus, there exists a balance point of *d* maximizing J.

The trends of defenders’ J observed are also very similar to the ones observed in attackers’ J in Fig 4. Due to the space constraint, we do not show the results here. The reason is that defenders basically follow attackers’ actions as they should recover the compromised nodes. However, in our results, defenders’ J is significantly lower than attackers’ because attackers consume more resource than defenders by taking actions to compromise user nodes.

**Fig 5:**
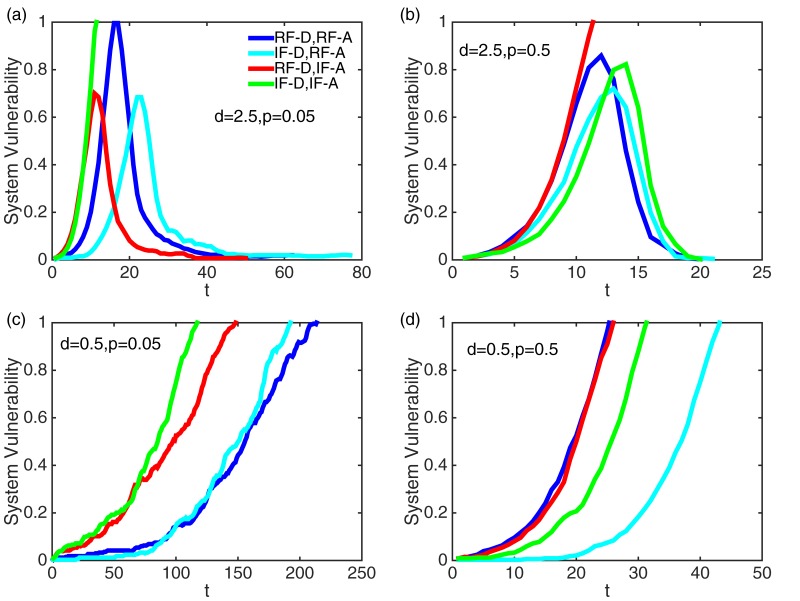
Effect of network temporality (*p*) and density (*d*) on system vulnerability (V(t)). (a) Plot of system vulnerability over time *t* under dense (*d* = 2.5) and low temporality (*p* = 0.05) networks. (b) Plot of system vulnerability over time *t* for dense (*d* = 2.5) and high temporality (*p* = 0.5) networks. (c) System vulnerability as a function of *t* for sparse (*d* = 0.5) and low temporality (*p* = 0.05) networks. (d) System vulnerability over time *t* under sparse (*d* = 0.5) and high temporality (*p* = 0.5) networks.

Lastly, Fig 5 shows how system vulnerability V(t) evolves over time under different network temporality and density. Comparing Fig 5(a) & 5(b) and 5(c) & 5(d) for a sparse network vs. a dense network, there exists a critical point that maximizes system vulnerability but three cases out of four do not experience system failure where V(t)=1 implies system failure. That is, although the system has a higher chance to be endangered by system vulnerability, it can survive over time by reducing the vulnerability. However, for a dense network, the system ultimately fails due to a high chance for nodes to be compromised by attackers. On the other hand, comparing Fig 5(a) & 5(c) and 5(b) & 5(d) for high temporality vs. low temporality, a longer time is taken to experience high vulnerability or failure under low temporality (i.e., Fig 5(a) & 5(c)) than under high temporality (i.e., Fig 5(b) & 5(d)).

## Conclusion

Given a cyber war game for a resource-constrained, temporal, distributed network, we studied how each party can win the game with minimum resource consumption. We devised two heuristic strategies in a greedy manner based on a node’s influence and resource level to maximize a win probability while minimizing resource consumption. We investigated the effect of the proposed heuristic strategies on performance metrics including a win probability, minimum resource consumption, and security vulnerability. We investigated how network temporality and density affect performance of the strategies.

We found that attackers’ influence-first (IF-A) strategy outperforms resource-first (RF-A) strategy under both sparse and dense networks across a wide range of network temporality. In addition, in a dense network, IF-A consumes less resource than RF-A. Network temporality helps attackers to win a game under a sparse network while it may deteriorate the win probability under a dense network due to a higher chance for them to be recovered by defenders. Although a higher win probability generates higher resource consumption, a certain point of node degree exists to maximize resource consumption. In addition, system vulnerability is significantly affected by network temporality and density because the network characteristics are critical for a node to reach its system goal state with minimum resource consumption.

Our work may raise the following open research questions: how can network temporality be described in real networks?; how can the proposed work be validated with a real network dataset?; If each node is modeled as an agent using game theory, how can Nash Equilibrium be identified in a cyber war game?; and how do heterogeneous network or node characteristics affect optimal strategies of attackers and defenders in a cyber war game?
